# Kartagener’s Syndrome: Situs Inversus, Chronic Sinusitis and Bronchiectasis

**DOI:** 10.5334/jbr-btr.955

**Published:** 2016-02-10

**Authors:** Robin Peters, Gonda de Jonge

**Affiliations:** 1 UMCG, NL

**Keywords:** Kartagener, situs inversus, chronic sinusitis, bronchiectasis, HRCT

## Case presentation

**Figure 1:**
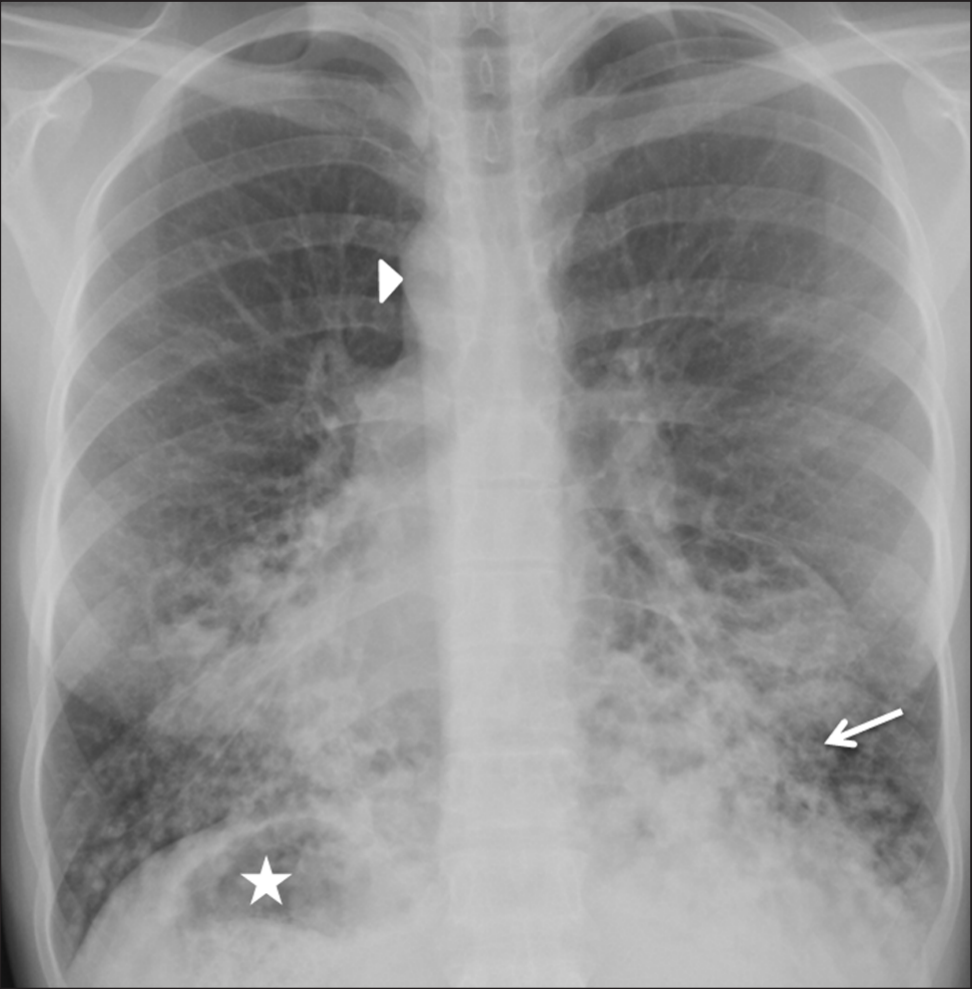


**Figure 2:**
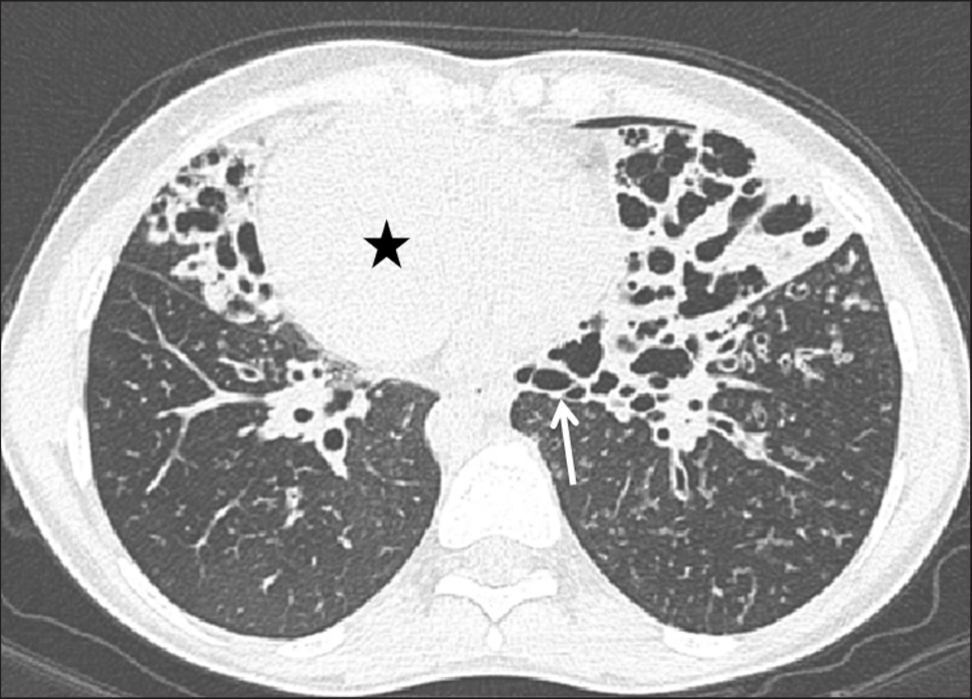


**Figure 3:**
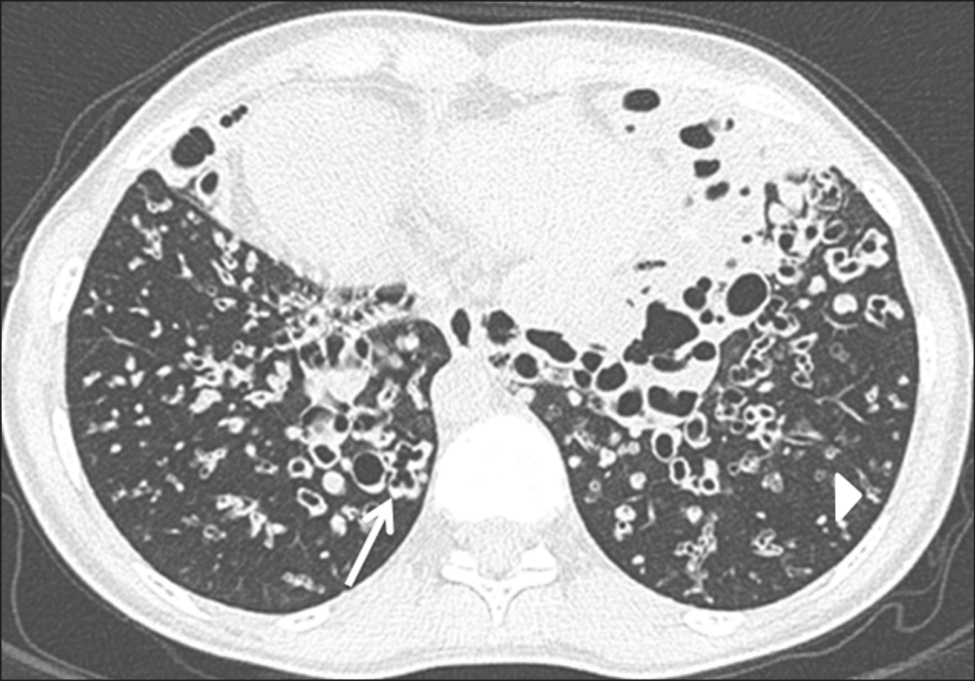


A 26-year-old woman with a nine-month history of dyspnea and productive cough was referred by the general practitioner to the Pulmonology department of our hospital. The patient, who recently returned from Syria, was treated for three weeks with antibiotics without clinical improvement. Physical examination revealed inspiratory wheeze without crepitations at the bases of both lungs. There was no fever. Anteroposterior chest radiograph showed dextrocardia, a right-sided aortic arch (arrowhead) and a right-sided gastric bubble (star) indicating situs inversus (Figure 1). Consolidations, mucus plugs and tram-tracks (arrow) in the lower zones of both lungs indicating bronchiectasis were visible. High resolution computed tomography (HRCT) of the lung demonstrated severe bilateral basilar bronchial wall thickening and bronchiectasis (Figures 2 and 3). Bronchiectasis were varicose and cystic with intrinsic air-fluid levels (arrow). A diffuse centrilobular nodular pattern (tree-in-bud pattern) was present in the left and right lower lobe (arrowhead). Situs inversus totalis was confirmed with a tri-lobed left lung, a bi-lobed right lung, dextrocardia (star), right-sided spleen and left-sided liver.

## Comments

Primary ciliary dyskinesia is an autosomal-recessive congenital disorder characterized by ciliary immotility resulting in abnormal mucociliary clearance. The most common clinical manifestations are recurrent bronchitis, pneumonia and sinusitis. Situs inversus is present in 50% of patients with dyskinetic cilia syndrome [1]. The triad of situs inversus, chronic sinusitis and bronchiectasis is called Kartagener’s syndrome which is a subgroup of primary ciliary dyskinesia. An incidence of one in 20,000–40,000 individuals is reported. Dyskinetic cilia syndrome is also associated with infertility in males and lowered fertility in females. Bronchiectasis are caused by recurrent infection and inflammation of the airways. HRCT of the lung is used to demonstrate and assess the severity of bronchiectasis. Bronchiectasis are classified into three morphologic types depending on the severity of bronchial dilatation (cylindrical, varicose and cystic) and wall abnormalities. As in our case, the distribution of bronchial wall thickening and bronchiectasis is bilateral and diffuse with a predilection for the lingula, middle and lower lobes. Mucus plugs within dilated airways and tree-in-bud opacities (centrilobular nodules) representing dilated and impacted centrilobular brionchioles are often present. Sometimes calcifications can be detected in the lumen of impacted bronchioles. Expiratory HRCT can reveal mosaic attenuation based on air trapping [1]. Patients are treated with antibiotics, supportive care and chest physiotherapy to prevent further damage.

## Competing Interests

The authors declare that they have no competing interests.
